# Towards a Better Understanding on How Cognitive Impairment Affects Pain

**DOI:** 10.3390/brainsci12020170

**Published:** 2022-01-27

**Authors:** Joukje M. Oosterman, Miriam Kunz

**Affiliations:** 1 Donders Institute for Brain, Cognition and Behaviour, Radboud University, 6525 GD Nijmegen, The Netherlands; 2 Department of Medical Psychology and Sociology, Medical Faculty, University of Augsburg, 86135 Augsburg, Germany; miriam.kunz@med.uni-augsburg.de

When judging whether someone is suffering from pain, the simplest and most reliable solution is to ask that person about it. However, this gold standard of assessing pain via self-report is less reliable in people suffering from cognitive impairment. In these individuals, their cognitive deficits limit their ability to reflect upon and to effectively communicate information about their pain. A widely recognized consequence is the heightened risk for inappropriate treatment (and in particular undertreatment) of pain in these individuals. To improve assessments and, consequently, treatments of pain in populations with cognitive impairments, over recent decades, there has been an upsurge in alternative pain assessment methods that mostly rely on nonverbal pain responses, such as facial expressions and body posture. Despite these efforts, several questions remain unanswered: How can we ensure the reliability of those assessment methods if the comparator, the gold standard of self-reported pain, is no longer reliable? What are appropriate cut-off points for differentiating between different levels of pain? How do the various neuropathological and cognitive changes in the diverse clinical populations relate to the outcomes of pain assessment methods? How aware are caretakers of the potential changes in pain experiences reported in cases of cognitive impairment, and what alternative pain assessment methods are available?

These examples illustrate the existing gap in the current literature on pain in individuals with cognitive impairments. At the same time, it highlights the complexity of assessing and interpreting signals of pain in patients with cognitive impairment. 

In this Special Issue, we take an important step forward by tackling the complex topic of pain assessment in individuals with impaired cognition from several angles. The articles included in this Special Issue focus on pain outcomes and pain responses in varies types and pathologies of cognitive impairments, ranging from individuals with intellectual disabilities [1,2,3] to older adults with varying degrees of cognitive impairments [4,5,6,7] and more severe forms of dementia [8]. Moreover, about half of the articles have a clear clinical perspective, with investigations focused on the reliability [9] and clinical cut-off scores [8] of observational pain assessment scales as well as reflections on the pain management and pharmacological treatment of pain in younger and older individuals with cognitive impairments [1,7]. Other articles applied controlled experimental pain stimuli to compare pain sensitivity between different pathologies of cognitive impairments [2,4].

Across all articles, it becomes evident that pain is still often overlooked in individuals with cognitive impairments and that observational pain assessment tools are necessary to improve this situation in these vulnerable individuals. The articles included also show promising findings, namely that these observational pain assessment scales allow for a reliable pain assessment that is relatively consistent across different regions in Europe, across different language versions, and across different pathologies [3,4,8,9]. Moreover, it also becomes apparent across articles that cognitive impairment is not necessarily associated with a decrease in pain responsiveness to noxious stimuli; in contrast, several articles point to an increase in pain responses in individuals with cognitive impairments [2,4,5]. Two of the articles in this special suggest that this increase in pain sensitivity—either measured via experimental or clinical pain assessment methods—seems to be associated with poor executive functioning [5,6]. This provides a target for potential treatment options, namely, to train executive functions in order to reduce pain vulnerability in individuals with cognitive impairments, at least when the cognitive impairment is not too severe. Thus, we hope to contribute to the field of pain assessment in individuals of impaired cognition by presenting this Special Issue in which we obtain better understanding of how cognitive impairment affects the processing of pain, of its clinical assessment and management, and of how assessment and management of pain can be improved in different clinical conditions.
